# MCAM: A Database to Accelerate the Identification of Functional Cell Adhesion Molecules

**DOI:** 10.4137/cin.s341

**Published:** 2008-03-31

**Authors:** Anguraj Sadanandam, Sudipendra Nath Pal, Joe Ziskovsky, Prathibha Hegde, Rakesh K. Singh

**Affiliations:** 1 Department of Pathology and Microbiology; 2 Information Technology Services Learning Environment/Internet Services, University of Nebraska Medical Center, Omaha, NE, U.S.A

**Keywords:** cell adhesion molecules, cancer, gene ontology, virtual gene expression, database, organ-specific homing, classification of cell adhesion molecules

## Abstract

In the post-genomic era, computational identification of cell adhesion molecules (CAMs) becomes important in defining new targets for diagnosis and treatment of various diseases including cancer. Lack of a comprehensive CAM-specific database restricts our ability to identify and characterize novel CAMs. Therefore, we developed a comprehensive mammalian cell adhesion molecule (MCAM) database. The current version is an interactive Web-based database, which provides the resources needed to search mouse, human and rat-specific CAMs and their sequence information and characteristics such as gene functions and virtual gene expression patterns in normal and tumor tissues as well as cell lines. Moreover, the MCAM database can be used for various bioinformatics and biological analyses including identifying CAMs involved in cell-cell interactions and homing of lymphocytes, hematopoietic stem cells and malignant cells to specific organs using data from high-throughput experiments. Furthermore, the database can also be used for training and testing existing transmembrane (TM) topology prediction methods specifically for CAM sequences. The database is freely available online at http://app1.unmc.edu/mcam.

## Introduction

Cell adhesion molecules (CAMs) are transmembrane (TM) glycoprotein receptors that help cells to undergo a selective process of cell-cell or cell-matrix interactions. By spanning the membrane, these molecules function as links between the intra- and extra-cellular environments of cells^1^. In addition to adherence, the direct cell-cell or cell-matrix interactions mediated by CAMs play vital roles in various cellular processes including embryogenesis, hematopoiesis, angiogenesis, cellular growth and differentiation, migration, invasion, tumorigenesis and metastasis.^1^–^3^

The current biochemical and cell biology techniques have helped in identification and characterization of several CAMs involved in various functions. However, in the post-genomic era, to accelerate the identification process a combination of high-throughput experimental and computational biology approaches is necessary. Unfortunately, the current resources for CAMs are dispersed in cyber space, and retrieval of all relevant information for CAMs individually from such disparate resources becomes highly inefficient and labor intensive. Therefore, a consolidated database for CAMs that provide sequences and information including gene expression profiles will facilitate research on CAMs. To our best knowledge, there is no such CAM-specific database available for adhesion molecules with cross-reference to other sources including virtual gene expression databases. This motivated us to curate a consolidated record of available CAM sequences including their annotated information.

## Design of the Database

### Data collection

**Figure 1:**
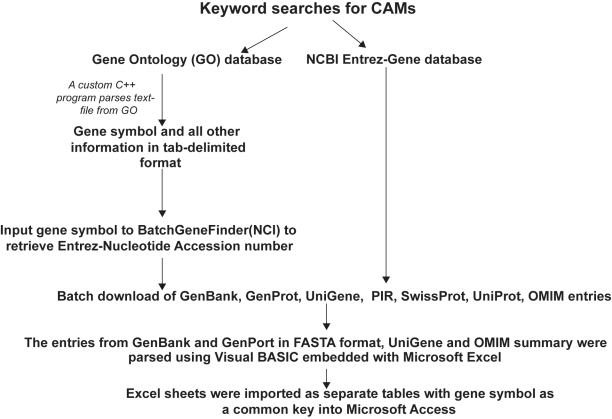
A schematic representation showing the construction of the MCAM database.

The MCAM database is a collection of functionally active CAMs curated from two different sources, the GO database and the Entrez Gene database. Construction of the database is shown in Figure 1. We searched the GO database at different periods of time (release dated 2003-10-01 to 2007-01-01) with keywords appropriate for CAMs that were selected from list of biological processes and molecular functions from the GO database. GO entries obtained from the above searches were downloaded and parsed using custom C++ scripts (available online) and used to populate the database. The gene symbols extracted were used as queries for Batch Gene Finder (http://cgap.nci.nih.gov/Genes/BatchGeneFinder) to obtain a list of GenBank^4^ accession numbers for the CAM entries. The accession numbers were used to obtain sequences from NCBI.

In addition to data from the GO database, the NCBI Entrez Gene database was searched using the keywords related to CAMs. Sequences from RefSeq database^5^ were obtained through the links from the Entrez Gene database entries. Similarly, entries from UniGene^6^ and Online Mendelian Inheritance in Man™ (OMIM) (Jan 2007)^7^ were downloaded following the respective links through the Entrez database. Protein sequences from Entrez,^8^ PIR (release 80)^9^ and UniProtKB/Swiss-Prot^10^ databases were also downloaded. The records for each entry were parsed and imported to Microsoft Excel using custom Visual Basic scripts (available online) embedded in Microsoft Excel.

For every CAM entry, the hyperlinks to GeneCards,^11^ GeneAtlas,^12^ CGAP — Gene Finder Tool^13^ and UniGene expression^14^ were also provided.

Using the gene symbols from mouse as queries, the human and rat CAMs were collected using Batch Gene Finder from CGAP and GeneInfoViz,^15^ respectively.

### Evaluation of data and classification of CAMs

The annotation of the Swiss-Prot entries such as ontologies, keywords and feature table viewer, were evaluated manually for the presence of terms related to CAMs. The entries which did not have CAM related annotations in UniProtKB/Swiss-Prot were validated manually for CAMs using PubMed literature searches. Entries not validated as CAMs were removed from the database. Furthermore, each CAM were classified in to integrins, immunoglobulin-like, cadherin and selectin using the UniProtKB/Swiss-Prot annotations and literature searches.

### Implementation

The data from Microsoft Excel were imported into Microsoft Access database and the Web interface was implemented using ColdFusion MX 7 and HTML 4.0. There are 22 tables in the database that include various data from different sources for mouse, human and rat CAMs (available online).

## Contents and Web Interface

### MCAM contents

The latest release (Version 3.0 dated 24 January, 2007) of the MCAM database includes information for CAMs from 298 GO database entries. The number of entries included in the database corresponding to GO terms from various database sources is listed in Table 1. The total number of entries included 863 from GenBank, 714 from GenPept, 874 from UniGene, 639 from Uni-ProtKB/Swiss-Prot, and 693 from PIR. The number of entries curated per species is summarized in Table 2. The number of entries differs due to the fact that the data sources such as PIR had redundant entries. Also, CAMs have been classified into superfamily of proteins and the number of entries in each class has been shown in Table 3.

### Web interface

The contents of the MCAM database can be searched using gene symbol, gene name or accession number. A search using gene name can be performed either by full text or partial text queries. The text queries are case insensitive and the searches using accession numbers include sources from GenBank, GenPept, UniGene, UniProtKB/Swiss-Prot, PIR or OMIM sources.

For example, a search for a limbic system associated membrane protein can be conducted using the gene symbol “lsamp” (case insensitive) or the gene name (either partial or full). The results will include gene symbol, gene name, and synonymous names of genes, nucleotide (GenBank), protein (GenPept), SPRT (UniProtKB/Swiss-Prot), PIR, OMIM, UniGene accession numbers and sequence data. Hyperlinks to NCBI–GenBank, GenPept, OMIM and UniGene, and UniProtKB/Swiss-Prot database entries are provided to retrieve further information about each CAM using the accession number as the query. Hyperlinks to GeneAtlas, GeneCards and NCBI Homologue database entries are provided with the gene symbol as the query. Literature search link is provided with PubMed using the gene symbol as a keyword. Virtual expression data for normal and cancer tissues and cell lines are provided through the Cancer Gene Anatomy Project (CGAP), and, normal adult and embryonic tissues through UniGene Expression hyperlinks. Functions of each CAM are provided through the GO database process and function.

## Discussion and Future Updates

The MCAM database is a web-based consolidated and searchable database of mammalian specific CAMs. It can be used for various bioinformatics and biological analyses including identifying CAMs involved in cell-cell interactions and homing of lymphocytes, hematopoietic stem cells and malignant cells to specific organs. It serves the research community by cataloguing information on CAMs available from many different databases.

With the growing amount of data from high-throughput technologies like phage display peptide library, our online MCAM database is critical for the identification of novel CAMs that are responsible for organ-specific homing of tumor cells. For example, local version of Basic Local Alignment Search Tool (BLAST)^16^ searches can be performed using any short oligonucleotides or peptides as queries against the CAM sequences available from the Download page as an input database. Once the CAMs are identified, the information including expression and functional profile of the proteins can be searched using the online MCAM database. We have identified 25 novel and known tumor-specific CAMs by BLAST searches utilizing the sequence data available from the MCAM database and seven amino acid peptides as queries.^17^

The MCAM database may also serve as a gene list for designing CAM specific oligonucleotide or cDNA probes for microarray experiments to examine the expression profiles of CAMs in various disease processes. Furthermore, the evolutionary conservation of each CAM gene within mouse, human and rat genomes can be studied using the MCAM database. Finally, the MCAM database can serve as a test or training dataset for identifying TM proteins, especially CAMs. Therefore, this database facilitates nucleotide and protein sequence analysis of CAMs assisting in CAM-specific genomics and proteomics experiments.

## Figures and Tables

**Table 1:** Number of entries from different database sources associated with GO terms.

**Gene ontology terms**	**GO**	**GenBank (FASTA)**	**UniGene**	**PIR**
Calcium dependent cell adhesion	12	6	8	25
Calcium independent cell adhesion	13	6	4	18
Cell adhesion	175	92	87	219
Cell-cell adhesion	50	23	19	52
Heterophilic cell adhesion	8	5	5	12
Homophilic cell adhesion	54	39	39	2
Positive regulation cell adhesion	2	1	1	2
Regulation cell adhesion	20	8	8	25

**Table 2:** Number of entries from different database sources representing mouse, human and rat is listed.

Sources	Mouse	Human	Rat
**GenBank**	502	312	49
**GenPept**	431	148	135
**PIR**	472	154	67
**UniProtKB/Swiss-Prot**	418	149	72
**UniGene**	610	184	80

**Table 3:** Superfamily classification of cell adhesion molecules and the number of entries in each class. The number of proteins whose classification is not known has also been shown.

Superfamily	Number of entries
Cadherin	47
Immunoglobulin superfamily	107
Integrin	21
Selectin	4
Not known	78
